# Genetic polymorphism of rs9564966 G > A on 13q22.1 predicts poor survival for Chinese patients with gastric cancer

**DOI:** 10.1002/cam4.1693

**Published:** 2018-12-08

**Authors:** Tingting Zhao, Xinying Huo, Jinfei Chen

**Affiliations:** ^1^ Department of Oncology Nanjing First Hospital Nanjing Medical University Nanjing China; ^2^ National Clinical Research Center of Kidney Diseases Jinling Hospital Nanjing University School of Medicine Nanjing China; ^3^ Collaborative Innovation Center for Cancer Personalized Medicine Nanjing Medical University Nanjing China

**Keywords:** 13q22.1, gastric cancer, rs9564966, single‐nucleotide polymorphism, survival

## Abstract

Two genomewide association studies on pancreatic cancer have identified a novel single‐nucleotide polymorphism of rs9564966 G > A on 13q22.1 region. However, the associations between the rs9564966 G > A polymorphism and the survival of Chinese patients with gastric cancer (GC) were unknown. In our present investigation, we adopted the Kaplan‐Meier plots, Cox regression analyses, and the log‐rank tests to explore the associations between rs9564966 G > A polymorphism and the prognosis of 911 Chinese patients with GC. Our results revealed that, compared with GG genotype, patients with GA + AA genotypes had poorer outcomes (HR = 1.348, 95% CI = 1.084‐1.675, *P *=* *0.007), especially in the subgroups of age ≤60 years, male, nondrinker, tumor size >5 cm, tumor site in Noncardia, intestinal‐type tumor, T3/T4 level depth of invasion, N1/N2/N3 level lymph node metastasis, no distant metastasis, III/IV level TNM stages, and no chemotherapy. Our findings suggested that the rs9564966 G > A polymorphism may be a potential biomarker to predict the survival of Chinese patients with GC.

## 1 INTRODUCTION

Gastric cancer (GC), the fourth most common and the second most deadly cancer worldwide, is particularly prevalent in developing countries.^1^ Specially, China possesses 47% of new global GC cases, with the highest mortality rate among all malignant cancers.^2^ Due to patients with GC at early stage are often asymptomatic, so they are often diagnosed at an advanced stage and miss the best treatment time, resulting in the 5‐year survival rate <25%.^3^, ^4^ Thus, the identification of additional biomarkers for early diagnosis and prognosis evaluation may play more crucial roles in the improvement of the treatment efficiency and the extension of survival time of patients with GC.^5^

The expression and function abnormal resulted from genetic changes (ie, deletion, amplification, and mutation) played more crucial roles in the initiation and progression of cancer.^6^ In particular, single‐nucleotide polymorphism (SNP) was the main force driving normal cells into cancer cells and promoting cell proliferation and invasion.^7^ Recently, two genomewide association studies (GWASs) on pancreatic cancer have identified a novel SNP of rs9564966 G > A.^8^, ^9^ The rs9564966 was located on chr 13q22.1 within a 600 kb nongenic region between Krüppel‐like factor 5 (KLF5) and Krüppel‐like 12 (KLF12), which may act as a blasting fuse influencing the expressions and functions of nearby genes through different mechanisms.^10^, ^11^, ^12^, ^13^, ^14^ KLFs were DNA‐binding transcriptional regulators involved in the regulations of various cellular processes, including development, differentiation, proliferation, and apoptosis.^15^ KLF5 (BTEB2, IKLF) was associated with numerous transcription factors (TFs) and played vital roles in reducing cell proliferation.^16^, ^17^ KLF12 (AP‐2rep), a novel Krüppel‐related zinc finger repressor, suppressed the expression of AP (Activator Protein)‐2a transcription factor through binding to the promoter region.^18^ Due to Rs9564966 was located between KLF5 and KLF12 on chr 13q22.1, so it may also play essential roles in the regulations of various cellular processes by influencing the functions of KLF5 and KLF12.

The abrogation of genes located on chromosome 13 was related to various cancers, including pancreatic, prostate, and breast cancers.^19^, ^20^, ^21^ Some studies reported that KLF5 acted as tumor suppressor to inhibit cell proliferation and function; KLF5 gene underwent frequent alteration and expression aberration in various cancer types.^22^, ^23^, ^24^, ^25^, ^26^ Other studies demonstrated that KLF12 knockdown significantly inhibited cell proliferation and overexpression of KLF12 promoted cell invasion.^11^ The levels of KLF12 mRNAs and proteins were obviously unregulated for cancer patients, revealing that KLF12 may confer growth advantage on cancer cells and play crucial roles in tumor progression.^11^ As a novel locus on 13q22.1 located between KLF5 and KLF12, whether SNP rs9564966 was associated with the survival of Chinese patients with GC was unknown. Therefore, we performed this study to explore the associations between rs9564966 polymorphism and the clinical prognosis of Chinese patients with GC.

## 2 PATIENTS AND METHODS

### 2.1 Study patients

All 919 GC patients who underwent surgery at the Yixing People's Hospital (Yixing, Jiangsu Province, China) between January 1999 and December 2006 were enrolled in our study. All the patients were histopathologically diagnosed and had no chemotherapy or radiotherapy before surgery. The patients’ clinical features were shown in Table 1. The survival time of GC patients was calculated from the date of surgery until death or to the last follow‐up in March 2009, and the median follow‐up time was 68.5 months. Tumors were classified into intestinal‐type or diffuse‐type by Lauren's criteria.^27^ The TNM stages were evaluated according to the TNM classification of the American Joint Committee on Cancer (AJCC cancer staging manual, seventh edition). All patients recruited in our study signed an informed consent on the use of clinical specimen for medical research. The institutional review board of Nanjing Medical University approved our present study. And all methods included in our investigation were performed in accordance with the relevant guidelines and regulations.

**Table 1:** The associations between the clinicopathological features and the survival of patients with gastric cancer

Variable	Patients, n = 911	Deaths, n = 420	MST (months)	Log‐rank *P*	HR (95% CI)^a^
Age (years)					
≤60	432	198	97	0.444	1.000
>60	479	222	62		1.077 (0.889‐1.305)
Sex					
Male	700	319	74	0.399	1.000
Female	211	101	63		1.100 (0.880‐1.376)
Tumor size (cm)					
≤5	566	237	74^b^	<0.001	1.000
>5	345	183	50		1.420 (1.171‐1.723)
Location					
Noncardia	600	280	70	0.4	1.000
Cardia	311	140	77		0.971 (0.749‐1.124)
Histological types					
Intestinal	388	150	77^b^	<0.001	1.000
Diffuse	523	270	50		1.457 (1.193‐1.779)
Differentiation^a^					
Well to moderate	298	126	80	0.510	1.000
Poorly	476	231	62		1.157 (0.931‐1.438)
Mucinous/signet‐ring cell	65	32	62		1.194 (0.810‐1.761)
Others	68	30	67^b^		0.991 (0.665‐1.477)
Depth of invasion^c^					
T1	177	57	85^b^	<0.001	1.000
T2	130	56	78		1.443 (0.997‐2.087)
T3	6	3	70		1.425 (0.446‐4.552)
T4	580	292	52		1.838 (1.382‐2.444)
Lymph node metastasis^c^					
N0	360	127	81^b^	<0.001	1
N1/N2/N3	540	286	46		1.770 (1.435‐2.182)
Distant metastasis					
M0	896	412	70	0.593	1
M1	14	7	47		1.224 (0.580‐2.583)
TNM stage					
I	236	79	83^b^	<0.001	1
II	197	78	71^b^		1.250 (0.914‐1.710)
III	449	246	39		2.008 (1.556‐2.590)
IV	21	11	40		2.011 (1.070‐3.780)
Chemotherapy					
No	608	281	74	0.457	1
Yes	299	136	60		1.081 (0.880‐1.327)

Variable	Patients, n = 911	Deaths, n = 418	MST (months)	Log‐rank *P*	HR (95% CI)^a^
Regimes					
L‐OHP	109	39	60	0.120	1
DDP	173	86	51		1.348 (0.921‐1.971)
Smoking					
Nonsmoker	836	389	65	0.336	1
Smoker	75	31	97		0.837 (0.580‐1.207)
Drinking					
Nondrinker	853	392	70	0.840	1
Drinker	58	28	77		1.040 (0.709‐1.526)

CI, confidence interval; HR, hazard ratio; MST, median survival time.

Adjusted for age and sex.

Mean survival time was provided when MST could not be calculated.

Information was not available for two patients.

### 2.2 Genotyping

Genomic DNA was extracted from paraffin sections of patients’ postoperative tissues by proteinase K digestion, isopropanol extraction, and ethanol precipitation.^28^ Rs9564966 G > A polymorphism was detected by SNaPshot technology based on an ABI fluorescence assay allelic discrimination method (Applied Biosystems, Forster City, CA). We designed the primers to anneal immediately adjacent to the nucleotide at the mutation site: rs9564966: forward, 5′‐ GAGAAAAGCCAGTTACATTACAGAACTTCC ‐3′; reverse, 5′‐ GAAAGATCACTAGGGCCCCTTCC ‐3′. The primers for extension were as follow: rs9564966, 5′‐ TTTTTGCCAGTTACATTACAGAACTTCCTTGATG‐3′. ABI3130 genetic analyzer was used to analyze the SNPs and the Genemapper 4.0 software (Applied Biosystems) was used to determine the genotypes. Genotyping assays were performed by two investigators independently in a blind fashion. Ten percent samples were randomly selected to validate genotypes and the results were 100% concordant.

### 2.3 Statistical analysis

The Student's *t* test for continuous data and the Pearson's chi‐square test for categorical variables were employed in estimating the associations between rs9564966 polymorphism and clinicopathologic parameters. When the median survival time (MST) could not be calculated, the mean survival time was chosen. Kaplan‐Meier plots and log‐rank tests were analysis by SPSS version 20.0 (SPSS Inc, Chicago, IL, USA). Univariate or multivariate Cox regression analysis was used to calculate the crude or adjusted hazard ratios (HRs) and the 95% confidence intervals (CIs). Moreover, we performed Cox stepwise regression analysis to assess the independent impacts of SNP or clinicopathologic features on the overall survival, with a significance level of *P *<* *0.05 for entering and *P *>* *0.10 for removal of the respective explanatory variables. All tests were two‐sided and *P *<* *0.05 was considered statistically significant.

## 3 RESULTS

### 3.1 Clinical characteristics of the study patients

A total of 919 patients were recruited in our investigation, and eight patients were removed due to lack of genotyping data. Finally, 911 patients with GC were enrolled and the clinical features of those patients were summarized in Table 1. All of the study subjects underwent surgery, and the median age was 62.0 years (range from 28 to 83 years old). Two hundred and eleven women and 700 men were included and 418 of them died during the follow‐up period of 119 months. Significant associations were founded between the survival time and the varieties in tumor size, histological types, depth of invasion, lymph node metastasis, and TNM stage (log‐rank < 0.001). In particular, patients with tumor size >5 cm (MST, 50 months) had a 42% obviously higher risk of death (HR = 1.420, 95% CI = 1.171‐1.723) compared with those with tumor size ≤5 cm (MST, 74 months). The intestinal‐type patients with GC (MST, 77 months) had a 45.7% significantly lower risk of death (HR = 1.457, 95% CI = 1.193‐1.779) than those diffuse‐type patients (MST, 50 months). T4 level depth of invasion (MST, 52 months) had an 83.8% significantly higher risk of death (HR = 1.838, 95% CI = 1.382‐2.444) than those T1 level depth of invasion (MST, 85 months). Patients with lymph node metastasis (MST, 46 months) had a 77% obviously higher risk of death (HR = 1.770, 95% CI = 1.435‐2.182) compared with those without metastasis (MST, 81 months). In addition, as the TNM stage increased, the risk of death also exhibited a significant increase (log‐rank *P *<* *0.001).

### 3.2 The polymorphism of rs9564966 located on 13q22.1 increased death risk

**Figure 1:**
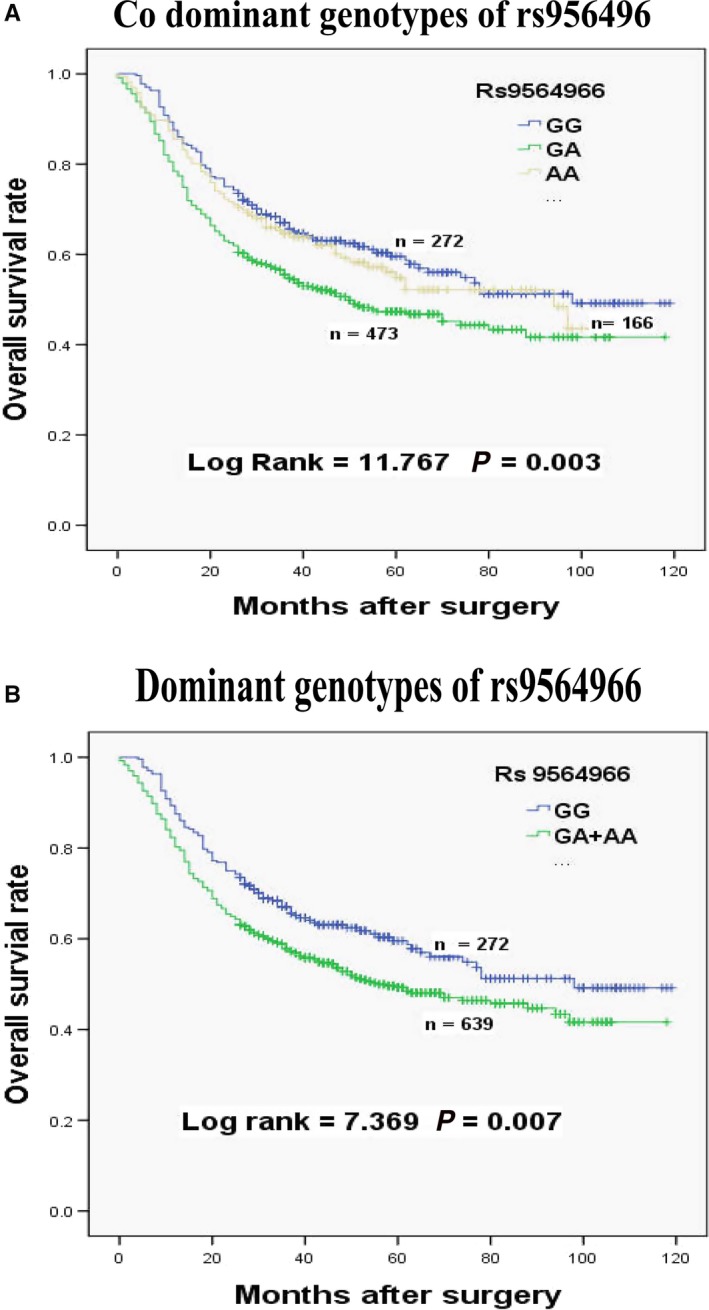
Kaplan‐Meier survival curves of rs9564966 polymorphism for the overall survival in patients with gastric cancer. A, Overall survival of codominant genotypes in patients with gastric cancer. B, Overall survival of dominant genotypes in patients with gastric cancer

Cox regression analyses were applied to analysis the relationships between rs9564966 polymorphisms and the survival of patients with GC in different genetic models, including codominant, dominant, and recessive models (Table 2). Our results firstly demonstrated that rs9564966 G > A polymorphism obviously increased the death risk of patients with GC. Compared with GG genotype, rs9564966 GA genotype significantly increased the death risks of patients with GC (HR = 1.444, 95% CI = 1.152‐1.811, *P *=* *0.003, Figure 1A). Similarly, the MST of GC patients with GA + AA genotypes was shortened from 98 to 56 months (HR = 1.348, 95% CI = 1.084‐1.675, *P *=* *0.007, Figure 1B) when compared to those with GG genotype.

**Table 2:** The associations between rs9564966 polymorphism and the overall survival of patients with gastric cancer

Genetic model	Genotypes	Patients	Deaths	MST (months)	Log‐rank *P*	HR (95% CI)^a^
Codominant model	GG	272	110	98	0.003	1
	GA	473	238	50		1.444 (1.152‐1.811)
	AA	166	72	94^b^		1.105 (0.821‐1.487)
Dominant model	GG	272	110	98	0.007	1
	GA/AA	639	310	56		1.348 (1.084‐1.675)
Recessive model	GG/GA	745	348	70	0.290	1
	AA	166	72	94		0.873 (0.677‐1.125)

CI, confidence interval; HR, hazard ratio; MST, median survival time.

a Adjusted for age and sex.

b Mean survival time was provided when MST could not be calculated.

### 3.3 Stratified analysis the associations between the polymorphism of rs9564966 G > A and prognosis of patients with GC

**Figure 2:**
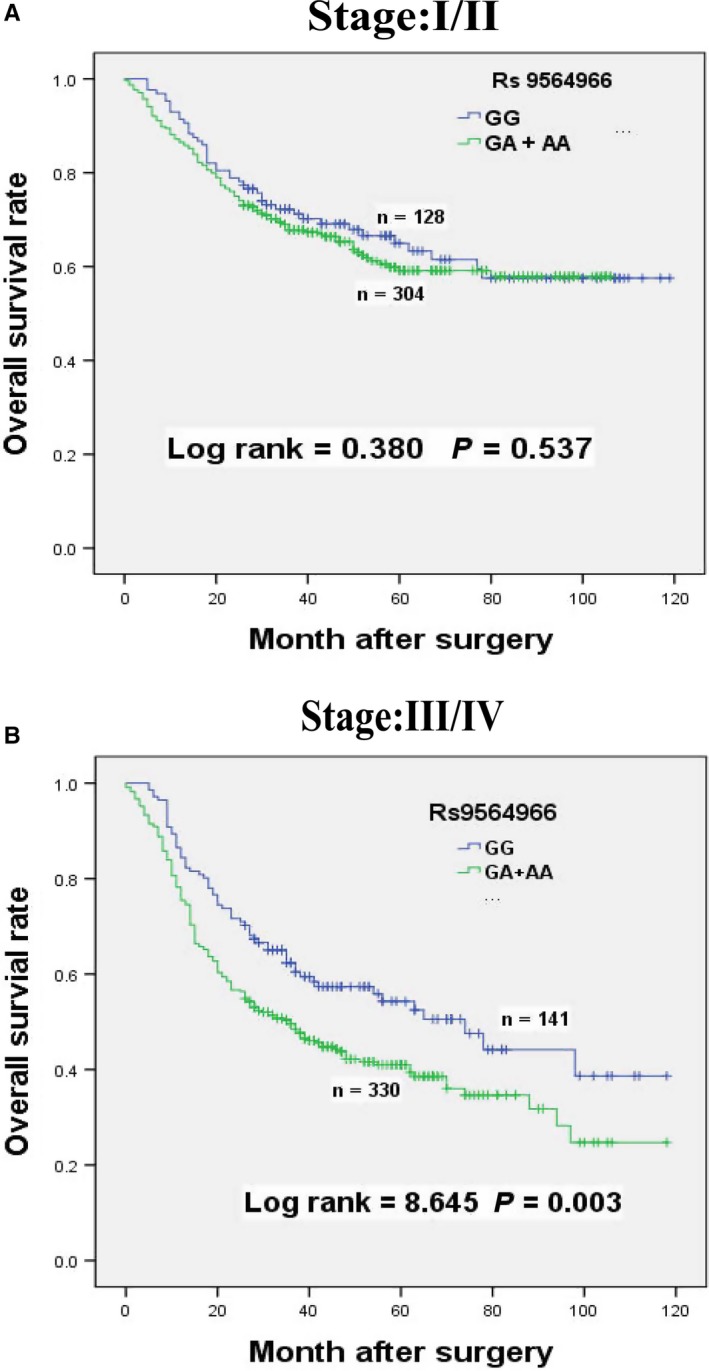
Overall survival curves of rs9564966 G > A polymorphism in dominant models with different TNM stages among patients with gastric cancer. A, Rs9564966 GA + AA were not associated with overall survival for stage I/II patients. B, Rs9564966 GA + AA were associated with poor overall survival for stage III/IV patients

Stratified analysis of age, sex, drinking wine, tumor size, tumor site, histological type, depth of invasion, lymph node metastasis, distant metastasis, TNM stage, and chemotherapy were performed to better assess the relationships between the polymorphism of rs9564966 G > A and GC patients’ survival (Table 3). Compared with GG genotype, the GA + AA genotypes were significantly related with poor survival of GC patients with age ≤60 years (HR = 1.407, 95% CI = 1.027‐1.927, *P *=* *0.032), male (HR = 1.291, 95% CI = 1.012‐1.646, *P *=* *0.038), nondrinker (HR = 1.305, 95% CI = 1.043‐1.634, *P *=* *0.019), tumor size >5 cm (HR = 1.507, 95% CI = 1.071‐2.122, *P *=* *0.017), and tumor site in Noncardia (HR = 1.345, 95% CI = 1.031‐1.755, *P *=* *0.027). Obvious negative associations were also obtained in the subgroups with tumor of intestinal type (HR = 1.608, 95% CI = 1.087‐2.378, *P *=* *0.016), T3/T4 level depth of invasion (HR = 1.323, 95% CI = 1.021‐1.714, *P *=* *0.032), N1/N2/N3 level lymph node metastasis (HR = 1.595, 95% CI = 1.221‐2.085, *P *<* *0.01), no distant metastasis (HR = 1.336, 95% CI = 1.072‐1.665, *P *=* *0.009), III/IV level TNM stages (HR = 1.518, 95% CI = 1.144‐2.015, *P *=* *0.003, Figure 2), and no chemotherapy (HR = 1.308, 95% CI = 1.005‐1.703, *P *=* *0.044).

**Table 3:** Stratified analysis of the associations between rs9564966 polymorphism and the overall survival of patients with gastric cancer

Variables	Genotypes (Dominant model)		MST (GG/GA + AA)	HR (95% CI)^a^	*P*_Heterogeneity_
	GG	GA/AA			
Total (N = 911)	272/110	639/310	98/56	1.348 (1.084‐1.675)	0.007
Age (years)					
≤60	133/53	299/145	78^b^/54	1.407 (1.027‐1.927)	0.032
>60	139/57	340/165	67/56	1.273 (0.942‐1.720)	0.113
Sex					
Male	222/91	478/228	98/56	1.291 (1.012‐1.646)	0.038
Female	50/19	161/82	77^b^/62	1.568 (0.950‐2.586)	0.073
Drinking					
Nondrinker	255/104	598/288	98/58	1.305 (1.043‐1.634)	0.019
Drinker	17/6	41/22	83^b^/39	2.265 (0.907‐5.658)	0.071
Tumor size (cm)					
≤5	174/67	392/170	78^b^/72^b^	1.218 (0.918‐1.617)	0.168
>5	98/43	247/140	77/43	1.507 (1.071‐2.122)	0.017
Tumor site					
Noncardia	180/74	420/206	76^b^/60	1.345 (1.031‐1.755)	0.027
Cardia	92/36	219/104	78/54	1.368 (0.935‐2.00)	0.103
Histological type^c^					
Intestinal type	106/32	282/118	86^b^/67^b^	1.608 (1.087‐2.378)	0.016
Diffuse type	166/78	357/12	63/48	1.259 (0.968‐1.639)	0.083
Depth of invasion					
T1/T2	95/29	212/84	87^b^/69^b^	1.461 (0.957‐2.229)	0.076
T3/T4	174/78	412/217	74/48	1.323 (1.021‐1.714)	0.032
Lymph node metastasis					
N0	101/35	259/92	83^b^/73^b^	1.097 (0.743‐1.619)	0.640
N1/N2/N3	168/72	372/214	78/36	1.595 (1.221‐2.085)	<0.01
Distant metastasis					
M0	266/108	630/304	98/60	1.336 (1.072‐1.665)	0.009
M1	5/1	9/6	45^b^/40	5.040 (0.598‐42.471)	0.100
TNM stage					
I/II	128/45	304/112	82^b^/72^b^	1.114 (0.788‐1.575)	0.537
III/IV	141/64	330/193	74/36	1.518 (1.144‐2.015)	0.003
Chemotherapy					
No	183/76	425/205	78/63	1.308 (1.005‐1.703)	0.044
Yes	87/33	212/103	69^b^/51	1.416 (0.956‐2.097)	0.079

CI, confidence interval; HR, hazard ratio; MST, median survival time.

a Adjusted for age and sex.

b Mean survival time was provided when MST could not be calculated.

Information was not available for two patients.

### 3.4 The independent impacts of SNP or clinicopathologic features on the overall survival after adjusting for other covariates

In the end, stepwise Cox regression analysis was used to analysis the associations between the rs9564966 G > A polymorphism, patients’ clinical features, demographic characteristics, and GC patients’ survival. As shown in Table 4, the rs9564966 G > A polymorphism and varieties in tumor size, lymph node metastasis, and depth of invasion were significantly related with the patient survival rates: *P *=* *0.003 for rs9564966 G > A dominant model: GA/AA vs GG; *P *<* *0.01 for tumor size: >5 cm vs ≤5 cm; *P *<* *0.01 for lymph node metastasis: N1/N2/N3 vs N0, and *P *=* *0.001 for depth of invasion: T3/T4 vs T1/T2, respectively.

**Table 4:** Stepwise Cox regression analysis on the survival of patients with gastric cancer

Variables	B	SE	HR	95% CI	*P* value
Age (>60 y vs ≤60 y)	0.08	0.1	1.083	(0.891‐1.317)	0.423
Sex (female vs male)	0.115	0.116	1.122	(0.894‐1.408)	0.319
Tumor size (>5 cm vs ≤5 cm)	0.352	0.101	1.422	(1.167‐1.732)	<0.01
Histological types (diffuse type vs intestinal type)	0.138	0.115	0.871	(0.696‐1.091)	0.229
Lymph node metastasis (N1/N2/N3 vs N0)	0.443	0.122	1.558	(1.226‐1.979)	<0.01
Depth of invasion (T3/T4 vs T1/T2)	0.375	0.118	1.455	(1.155‐1.834)	0.001
TNM stage (III/IV vs I/II)	0.261	0.235	1.298	(0.891‐2.059)	0.267
Domoniant model (GA/AA vs GG)	0.337	0.144	1.401	(1.120‐1.752)	0.003

B, relative risk rate; CI, confidence interval; HR, hazard ratio; SE, Standard error.

## 4 DISCUSSION

In our present study, we firstly investigated the clinical effects of rs9564966 G > A polymorphism on the prognosis of Chinese patients with GC. The dominant genotypes GA + AA were obviously related with poor survival of patients with GC, especially in the subgroups of age ≤60 years, male, nondrinker, tumor size >5 cm, tumor site in noncardia, intestinal‐type tumor, T3/T4 level depth of invasion, N1/N2/N3 level lymph node metastasis, no distant metastasis, III/IV level TNM stages, and no chemotherapy.

Two GWASs on pancreatic cancer have discovered a novel SNP rs9564966 G > A in gene deserts of 13q22.1 region, which may act as a blasting fuse disrupting the function of nearby normal gene through different mechanisms.^12^, ^13^, ^14^ SNP rs9564966 was located on the first sub‐band region of 13q22 between transcription factors KLF5 and KLF12.^10^, ^11^ KLF5 was associated with numerous transcription factors and played vital roles in reducing development, proliferation, and transformation of different cells.^16^, ^17^ And KLF12 suppressed the expression of the AP‐2a transcription factor through binding to the promoter region and overexpression of KLF12 promoted cell invasion.^11^, ^18^ So, the polymorphism of rs9564966 G > A on 13q22.1 region may also play a vital role in the initiation and progression of GC by influencing the functions of KLF5 and KLF12.

Several studies showed that the expression of KLF5 was downregulated or absent in patients with GC,^29^ and similar reduction was also occurred in other cancers, including prostate, breast, esophageal, and intestinal cancer.^22^, ^24^, ^25^ Nakamura et al^11^ showed that KLF12 level was remarkably upregulated for patients with GC and the knockdown of KLF12 induced growth arrest and significantly inhibited GC cell proliferation and invasion. For the SNP of rs9564966 G > A, recent studies demonstrated that the polymorphism of rs9564966 G > A significantly increased the risk of pancreatic cancer,^8^, ^9^, ^19^ which was similar to our results in patients with GC. Our finding indicated that patients with GA + AA genotypes had poorer prognosis than those with GG genotype (HR = 1.348, 95% CI = 1.084‐1.675, *P *=* *0.007, Figure 1). The explanation for this fact may be that the SNP of rs9564966 G > A affected the expressions and functions of the nearest upstream and downstream genes, including KLF5 and KLF12.^3^ The polymorphism of rs9564966 G > A may reduce KLF5 expression and improve KLF12 level through different mechanisms, exerting vital roles in tumor progression. Reduced expression of KLF5 would decrease the growth inhibitory function and the improved expression of KLF12 could promote cell proliferation and invasion.^11^, ^29^

Stratified analysis of the SNP with various subgroups based on clinical features was vital to identify the potential prognostic markers for patients with GC. Our findings observed that, compared with the GG genotype, the rs9564966 GA + AA genotypes could significantly decrease the survival time of GC patients with age ≤60 years, male, nondrinker, tumor size >5 cm, intestinal type of cancer, T3/T4 level depth of invasion, N1/N2/N3 level lymph node metastasis, no distant metastasis, and III/IV level TNM stage. Furthermore, GA + AA patients without chemotherapy had poorer prognosis than those with GG genotype, and no similar result was found among patients underwent chemotherapy. The expression changes of KLF5 and KLF12 genes affected by rs9564966 polymorphism may be also the possible explanations of this phenomenon.^12^, ^13^, ^14^

Nevertheless, several limitations were existed in our present study. First, we only examined SNP of rs9564966 located on 13q22.1, but more polymorphisms at other loci and the synergistic effects of those genes interacted with each other should be further tested. Second, the maximum follow‐up time of the patients recruited in our study was 119.0 months, so more studies with longer follow‐up time were warranted to be carried out to validate our results. Third, our present study was carried out in Chinese, whether similar results also existed among different populations should be tested in other researches. Last, *H. pylori* infection played a crucial role in the process of gastric carcinogenesis. But, the status of *H. pylori* infection was not included in our study for lack of clinical information.

In summary, we observed that rs9564966 G > A polymorphism was associated with the poor survival of patients with GC, suggesting that the polymorphism may be a useful potential biomarker for the prognosis of Chinese patients with GC.

## CONFLICT OF INTEREST

The authors report no conflict of interest in this work.

## INFORMED CONSENT

All the patients enrolled in our investigation provided ethics statements and a statement of informed consent.
